# Nuclear Factor of Activated T Cells Regulates the Expression of Interleukin-4 in Th2 Cells in an All-or-none Fashion*

**DOI:** 10.1074/jbc.M114.587865

**Published:** 2014-07-17

**Authors:** Juliana Köck, Stephan Kreher, Katrin Lehmann, René Riedel, Markus Bardua, Timo Lischke, Manja Jargosch, Claudia Haftmann, Hanna Bendfeldt, Farahnaz Hatam, Mir-Farzin Mashreghi, Ria Baumgrass, Andreas Radbruch, Hyun-Dong Chang

**Affiliations:** From the German Rheumatism Research Center Berlin, a Leibniz Institute, Charitéplatz 1, 10117 Berlin, Germany

**Keywords:** Calcineurin, Cytokine, NFAT Transcription Factor, T-cell, T-cell Receptor (TCR)

## Abstract

Th2 memory lymphocytes have imprinted their *Il4* genes epigenetically for expression in dependence of T cell receptor restimulation. However, in a given restimulation, not all Th cells with a memory for IL-4 expression express IL-4. Here, we show that in reactivated Th2 cells, the transcription factors NFATc2, NF-kB p65, c-Maf, p300, Brg1, STAT6, and GATA-3 assemble at the *Il4* promoter in Th2 cells expressing IL-4 but not in Th2 cells not expressing it. NFATc2 is critical for assembly of this transcription factor complex. Because NFATc2 translocation into the nucleus occurs in an all-or-none fashion, dependent on complete dephosphorylation by calcineurin, NFATc2 controls the frequencies of cells reexpressing *Il4*, translates analog differences in T cell receptor stimulation into a digital decision for *Il4* reexpression, and instructs all reexpressing cells to express the same amount of IL-4. This analog-to-digital conversion may be critical for the immune system to respond to low concentrations of antigens.

## Teaser

**Background:** Not every T helper type 2 (Th2) lymphocyte imprinted to express interleukin-4 (IL-4) does so when activated.

**Results:** Preventing nuclear translocation of the nuclear factor of activated T cells (NFAT) reduces the number of Th2 lymphocytes reexpressing IL-4.

**Conclusion:** NFAT is the limiting factor determining digital IL-4 expression in Th2 lymphocytes.

**Significance:** This might help us to understand the regulation of immunopathology in allergy and asthma.

## Introduction

T helper (Th)^2^ lymphocytes regulate immune responses by expression of cytokines instructing themselves and other cells to qualified reactions. Different cytokines are expressed by different lineages of Th cells, to adapt immune responses to the diversity of pathogens. Differentiation of activated Th cells into a particular lineage is induced by costimulatory signals and determined by lineage-determining transcription factors. T-bet, GATA-3, and RORγt determine the Th1, Th2, and Th17 lineages, respectively (reviewed in Ref. 1). Lineage-determining master transcription factors are both essential and sufficient for the differentiation of Th cells into a distinct lineage.

Expression of GATA-3 is under the control of a distal promoter responsive to T cell receptor stimulation, and of a proximal promoter responsive to GATA-3 itself and to STAT6, the signal transducer of the receptor for the cytokine interleukin-4 (IL-4) (2, 3). Once induced, GATA-3 expression is stabilized by a positive feedback loop (4, 5). IL-4 is the signature cytokine of Th2 lymphocytes. GATA-3 is critical for the epigenetic imprinting of IL-4 for reexpression in reactivated Th2 cells (6). GATA-3 binds to a conserved intronic regulatory element (CIRE) in the first intron of the *Il4* gene and induces its demethylation, which correlates with its imprinting for reexpression (7). GATA-3 has been described to block methyl CpG binding domain protein-2, which links DNA methylation to silent chromatin (8). Other regulatory elements of the *Il4* gene include the hypersensitivity site Va (9) important for lineage-specific binding of NFAT to the *Il4* locus and a locus control region (LCR) within the *Rad50* gene upstream of the *Il4* gene (10). In addition to GATA-3, some other transcription factors participating in the transcriptional control of the *Il4* gene such as STAT6 (11), Brahma-related gene 1 (Brg1) (12), and Creb-binding protein CBP/p300 (13) have the ability to recruit histone acetyltransferases and block DNA methyltransferases. Th2 reexpress their imprinted *Il4* gene upon restimulation of the T cell receptor (14), however, not all of them.

A substantial fraction of Th2 cells will not reexpress *Il4* in a given restimulation. This is not due to an insufficient imprinting of the gene because the very same cells can reexpress the *Il4* gene in later restimulations, with similar efficacy as their sister cells in the original restimulation (15). The reason for the failure of a Th2 cell to reexpress *Il4* in a given restimulation could be either a rate-limiting, stochastic availablility of transcription factors controlling *Il4* expression in the nucleus, leading to monoallelic expression of the *Il4* gene, with some cells not expressing it at all (16). Alternatively, one transcription factor could control the assembly of the *Il4* transcriptional complex in an all-or-none fashion. This has been demonstrated for the control of reexpression of the cytokine IL-2, which is dependent on translocation of NFATc2 into the nucleus (17). This translocation is dependent on complete dephosphorylation of NFATc2 at 13 positions by calcineurin (18), a reaction of second order, resulting in an all-or-none translocation of NFATc2 into the nucleus in individual Th2 cells. In addition, dephosphorylation at serine residues in the N-terminal transactivation domain is required for transcriptional activation.

Here, we show that in restimulated Th2 cells NFATc2 controls the reexpression of *Il4* in an all-or-none fashion. NFATc2 translocation into the nucleus is required for assembly of the transcription factor complex at the *Il4* promoter, which occurs only in IL-4-expressing Th2 cells, upon restimulation of the T cell receptor (TCR). Modulation of TCR signaling strength by graded inhibition of NFAT results in decreasing frequencies of IL-4-expressing Th2 cells. The amount of IL-4 produced by expressing cells is not affected. Thus, in Th2 cells NFAT serves as a molecular switch that translates graded differences in TCR signal strength into a digital decision to express IL-4 or not.

## EXPERIMENTAL PROCEDURES

### 

#### 

##### Mice

BALB/c, C57BL/6, OVA-TCRtg/tg DO11.10 (kind gift of Dennis Y. Loh and Kenneth Murphy, Washington University School of Medicine, St. Louis, MO), and OT-II mice were bred under specific pathogen-free conditions in our animal facility. The mice were sacrificed by cervical dislocation. All animal experiments were performed in accordance with institutional, state, and federal guidelines.

##### Antibodies

All antibodies used in these experiments were either conjugated in-house or purchased as indicated. Anti-IL-4 (11B11), anti-IL-12 (C17.18), anti-IFNγ (AN17.18.24), and anti-CD4 (GK1.5) antibodies were purified from hybridoma supernatants at the German Rheumatism Research Center and used at 10 μg/ml final concentration. FITC-conjugated anti-IFNγ (AN18.17.24; BD Pharmingen, Heidelberg, Germany), phycoerythrin-conjugated anti-IL-4 (11B11; BD Pharmingen, and BVD4–1D11; Miltenyi Biotec, Bergisch Gladbach, Germany) were used for intracellular cytokine staining. For the chromatin immunoprecipitation, the following antibodies were used: anti-c-MAF, anti-RNA polymerase II, anti-p300 and anti-STAT6 (polyclonal rabbit IgG; Santa Cruz Biotechnology, Heidelberg, Germany), anti-NFATc2 and -NFATc1 (polyclonal rabbit IgG; ImmunoGlobe Antikörpertechnik GmbH, Himmelstadt, Germany), anti-GATA-3 (mouse monoclonal; Santa Cruz Biotechnology), anti-NF-kB (polyclonal goat IgG; Santa Cruz Biotechnology), anti-Brg1 (rabbit antiserum; Merck-Millipore, Darmstadt, Germany). For the image cytometry, anti-NFATc2 (rabbit monoclonal, clone D4B1, Cell Signaling Technology, Leiden, The Netherlands) and donkey anti-rabbit IgG (coupled to Alexa Fluor 647, Molecular probes A31573, Darmstadt, Germany) were used.

##### In Vitro Th Cell Differentiation

CD4^+^CD62L^+^ cells from 6–8-week-old DO11.10 or OT-II mice were isolated and differentiated into Th1 and Th2 lineages as described (19). In short, for Th1 differentiation, cells were stimulated in the presence of recombinant IL-12 (5 ng/ml; R&D Systems, Wiesbaden, Germany) and anti-IL4 (11B11) antibody for 6 days. For Th2 differentiation, cells were stimulated in the presence of IL-4 (100 ng/ml, culture supernatant of HEK293T cells transfected with murine IL-4 cDNA), anti-IL12 (C17.8), and anti-IFNγ (AN18.17.24) antibodies.

##### Isolation of Viable IL-4 Secreting Cells

Viable IL-4 secreting cells were isolated as described previously (14). The secreted IL-4 was detected with an anti-IL4 phycoerythrin-conjugated antibody (Miltenyi Biotec). The IL-4 producing cells and the IL-4 non-producing cells were separated by MACS using anti-phycoerythrin microbeads (Miltenyi Biotec). After sorting, the purity of the sort was confirmed with a FACSCalibur (BD Biosciences).

##### Chromatin Immunoprecipitation

Cells were harvested at the indicated time points and fixed with 1% formaldehyde for 10 min at room temperature. The chromatin immunoprecipitation assay was performed as described previously (19). The following primers were used: *Il4* promoter up, 5′-GGCCCAGAATAACTGACAATCT-3′ and *Il4* promoter down, 5′-GCAATGCTGGCAGAGGTC-3′; CIRE up, 5′-CACTTGAGAGAGATCATCGG-3′ and CIRE down, 5′-CCACCTCTCTAGCAACTCAG-3′; *Il4* hypersensitivity site (HSS) Va up, 5′-TTGGGTTCTCAGTCCAACAGA-3′ and *Il4* HSS Va up, 5′-CCAGGGCACTTAAACATTGC-3′; CNS1 up, 5′-GGGAGTTTCTTAGGCCCTCT-3′ and CNS1 down, 5′-CCCCCTCTCACTGTGAAAAC-3′; LCRRad50 up, 5′-CCACACACTGGGATGTGTAGCTCA-3′ and LCRRad50 down, 5′-AGACCCAGCTCCTCAGAAGGTAGT-3′; and *Ifng* promoter up, 5′-TTTCAGAGAATCCCACAAGAATG-3′ and *Ifng* promoter down, 5′-TCGGGATTACGTATTTTCACAAG-3′.

##### Intracellular Cytokine Staining

For intracellular cytokine staining, the cultured Th cells are harvested and restimulated with 10 ng/ml phorbol 12-myristate 13-acetate (PMA) and 1 μg/ml ionomycin (Sigma) for 2 h followed by additional 2 h in the presence of 5 μg/ml brefeldin A (Sigma Chemicals). The cells were washed with PBS and fixed with 2% formaldehyde (Merck-Millipore). The cell membrane was permeabilized with 0.5% sapinon in PBS/BSA for intracellular staining. The staining was measured with a FACSCalibur (BD Biosciences) or with a MACSQuant Analyzer (Miltenyi Biotec), and the data were analyzed using FlowJo (Treestar). For calcineurin/NFAT inhibition, cyclosporin A, BTP1 (3,5-bistrifluoromethyl pyrazole), or 11R-VIVIT (Calbiochem) was given to the cells at the indicated concentrations 15 min prior to addition of PMA and ionomycin. For siRNA-mediated inhibition of NFATc2, Accell siRNA (A-054724-16; Dharmacon, Lafayette, CO) specific for NFATc2 and control siRNA (D-001910-03-05; Dharmacon) were used as described in Ref. 20. Knockdown efficiency was determined by RT-PCR using the following primers: NFATc2 up, 5′-GGTTGCTCCTCTGCCCGCAG-3′ and NFATc2 down, 5′-TTGGAGGGGATCCCGCAGGG-3′.

##### Image Cytometry

Th2 cells were harvested and restimulated with PMA/ionomycin for 3 h in the presence of 7 nm cyclosporin A (CsA). The cells were fixed with 1% formaldehyde. Cytokine staining was performed in 0.5% saponin. NFATc2 staining was done in Foxp3 staining buffer (eBioscience). Staining was analyzed using the Imagestream MKII (Amnis Merck-Millipore). For nuclear staining, DAPI was added before analysis. Data analysis was performed using the IDEAS software (Amnis). Nuclear localization of NFATc2 was determined by “similarity” of NFATc2 and DAPI on a per cell basis. The similarity score is a log-transformed Pearson's correlation coefficient of the pixel values of the DAPI and NFATc2 staining.

## RESULTS

### 

#### 

##### Assembly of the Activating Transcription Factor Complex at the Il4 Locus in Th2 Cells Is Dependent on T Cell Receptor Stimulation

**FIGURE 1.**
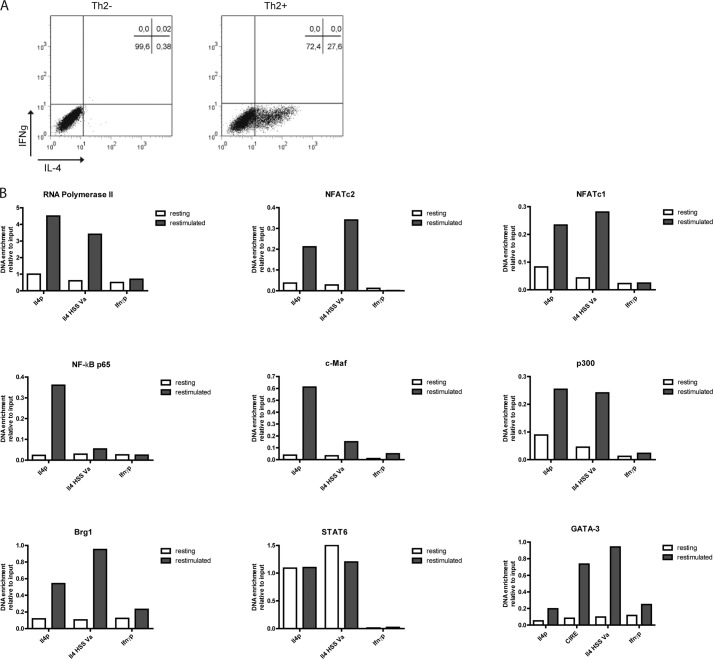
**The binding of transcription factors to the Il4 locus is dependent on restimulation.** *A*, Th2 cells polarized in the presence of recombinant IL-4, anti-IL12, and anti-IFNγ antibodies for 12 days were restimulated with PMA and ionomycin for 3 h and then stained intracellularly for IL-4 and IFNγ expression. *B*, the Th2 cells were either fixed in a resting state or after restimulation. ChIP was performed against RNA polymerase II, NFATc2, NFATc1, NF-κBp65, c-maf, CBP/p300, Brg1, STAT6, and GATA-3 and probed for binding regions in the *Il4* gene. Binding to the *Ifn*γ promoter was used as negative control. Representative result of three independent experiments.

We generated Th2 cells by stimulating naive CD4^+^CD62L^+^ T cells from ovalbumin-specific T cell receptor transgenic DO11.10 mice with OVA_323–339_ for 12 days in the presence of recombinant IL-4 and antibodies blocking IFN-γ and IL-12. On day 6, fresh antigen-presenting cells, antigen, IL-4, and antibodies were added. On day 12, the resting Th2 cells were either fixed directly or restimulated with PMA and ionomycin for 3 h and used for chromatin immunoprecipitation (ChIP) to assay for binding of RNA polymerase II and the transcription factors NFATc2, NFATc1, NF-κB p65, c-Maf, p300, Brg1, STAT6, and GATA-3 to the *Il4* promoter and the *Il4* HSS Va. Binding of GATA-3 to CIRE was also assayed. Binding to the *Ifn*γ promoter was used as negative control. In resting Th2 cells, in which no IL-4 is detectable by intracellular cytokine staining (Fig. 1*A*), none of the transcription factors with the exception of STAT6 bound to any of the regions tested (Fig. 1*B*). STAT6 bound to both the *Il4* promoter and *Il4* HSS Va to the same degree in unstimulated and restimulated cells. In restimulated Th2 cells, all transcription factors analyzed bound to the *Il4* promoter, except for GATA-3, which does not have a binding site there. NFATc2, NFATc1, p300, Brg1, and GATA-3 also bound to the *Il4* HSS Va. GATA-3 also bound to the CIRE. Thus, the assembly of TCR dependent and independent transcription factors at the *Il4* gene of Th2 cells is dependent on TCR stimulation, *i.e.* the activation of one or more TCR-dependent transcription factors.

##### Coordinated Assembly of Transcription Factors at the Il4 Locus

**FIGURE 2.**
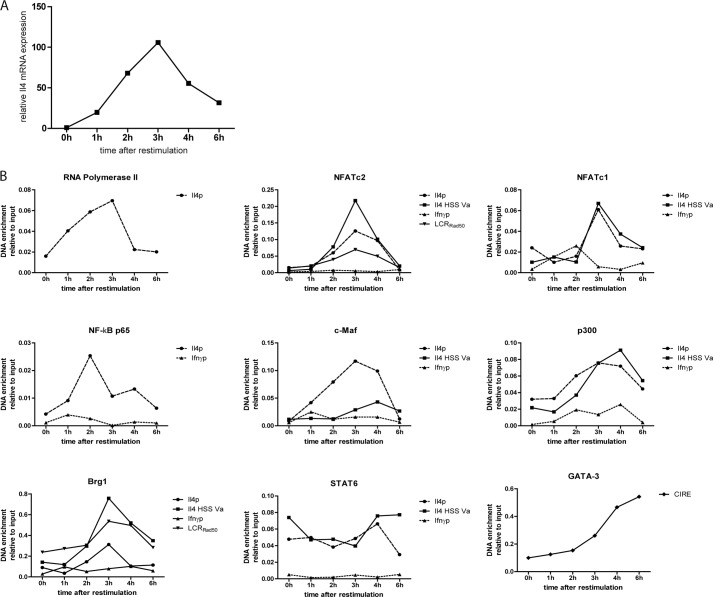
**The binding of transcription factors to the Il4 locus occurs in a coordinated fashion.** *A*, Th2 cells polarized for 12 days were restimulated, and the Il4 mRNA expression was quantified every hour for 6 h. *B*, the Th2 cells were restimulated, and aliquot was fixed every hour. ChIP was performed against the indicated transcription factors to determine the binding kinetic of the factors to selected regulatory regions in the *Il4* locus. Binding to the *Ifn*γ promoter was used as negative control. Shown are the representative results of two independent experiments.

To determine the kinetics of transcription factor assembly to the *Il4* locus, we fixed *in vitro*-generated Th2 cells before restimulation (0 h) and 1, 2, 3, 4, and 6 h following restimulation with PMA/ionomycin. Activated Th2 cells showed detectable levels of IL-4 mRNA already after 1 h and reached a maximum expression at 3 h, after which it declined again (Fig. 2*A*). IL-4 protein expression follows a similar time course (14). Binding of RNA polymerase II to the *Il4* promoter reached a maximum after 3h, with an abrupt drop to baseline levels after 4 h (Fig. 2*B*). The transcription factors NFATc2, NFATc1, c-MAF, p300, and Brg1 reached their maximal binding to the *Il4* promoter after 3 h. The transcription factors analyzed bound with similar kinetics also to the *Il4* HSS Va. No significant binding to the *Ifn*γ promoter could be detected at any time point. STAT6 bound to the *Il4* promoter and *Il4* HSS Va at all time points tested. NFATc2 and Brg1 also bound to the locus control region, located in the *Rad50* gene (LCR_Rad50_), reaching maximum binding after 3 h. GATA-3 binding to the CIRE increases after PMA/ionomycin restimulation and continues to increase until 6 h after the onset of restimulation, the end of the period of observation. The kinetics of transcription factor assembly at the *Il4* gene indicates the coordinated, interdependent assembly of all factors.

##### The Transcription Factor Complex Assembles at the Il4 Locus in IL-4 Expressing Th2 Cells but Not in IL-4-non-expressing Th2 Cells

**FIGURE 3.**
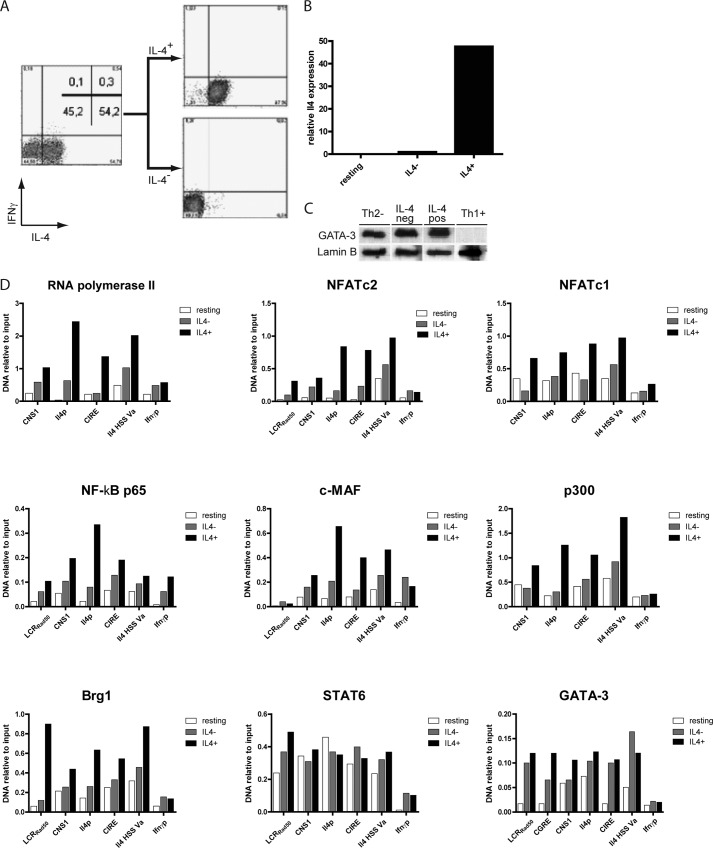
**The assembly of a transcription factor complex occurs only in Il-4 expressing Th2 cells.** *A*, Th2 cells polarized for 12 days were restimulated and sorted for IL-4 expression using the IL-4 cytokine secretion assay. *B*, Th2 cells isolated according to IL-4 secretion were lysed, and the *Il4* mRNA expression was quantified in resting Th2 cells, Th2 cells not secreting IL-4, and Th2 cells secreting IL-4. *C*, Th2 cells isolated according to IL-4 secretion were lysed, and the GATA-3 protein expression was quantified by Western blot in resting Th2 cells, Th2 cells not secreting IL-4, Th2 cells secreting IL-4, and restimulated Th1 cells. *D*, Th2 cells polarized for 12 days were either fixed in a resting state or following restimulation and separation into IL-4 secreting and IL-4 non-secreting Th2 cells. ChIP was performed against the indicated transcription factors and probed for binding to regulatory regions in the *Il4* locus. Binding to the *Ifn*γ promoter was used as negative control. Shown are the representative results of three independent experiments.

Th2 cells were restimulated with PMA/ionomycin, and the IL-4 expression was determined (Fig. 3*A*). Of the Th2 cells, 55% expressed IL-4, whereas 45% did not express any detectable IL-4. IL-4-expressing and non-expressing Th2 cells were physically separated to >95% purity, using the IL-4 cytokine secretion assay, which we had developed earlier (14). IL-4 protein expression correlated with IL-4 mRNA expression as analyzed by quantitative PCR in the sorted populations (Fig. 3*B*). Both the IL-4 expressing and non-expressing Th cells expressed equal amounts of GATA-3, qualifying both fractions as *bona fide* Th2 cells (Fig. 3*C*).

Relative to the binding of the transcription factors to the *Ifn*γ promoter, no significant binding to any of the *Il4* gene regions analyzed from IL-4-non-expressing cells was observed for NF-κB p65, c-Maf, and RNA polymerase II. NFATc2, NFATc1, p300, and Brg1 did not bind to the promoter and CIRE regions, whereas STAT6 and GATA-3 did (Fig. 3*D*). STAT6 and GATA-3 also bound to the CIRE, LCR_Rad50_, and HSS Va regions of Th2 cells not expressing IL-4 (Fig. 3*D*). For all regions of *Il4* analyzed, significantly more RNA polymerase II, NFATc2, NFATc1, NF-κB p65, c-Maf, p300, and Brg1 was detected in IL-4 expressing *versus* non-expressing cells. Thus, the transcription factor complex, with the exception of GATA-3 and STAT6, efficiently assembles only at *Il4* genes of IL-4-expressing Th2 cells.

##### Calcineurin Digitalizes IL-4 Expression in Th2 Cells

**FIGURE 4.**
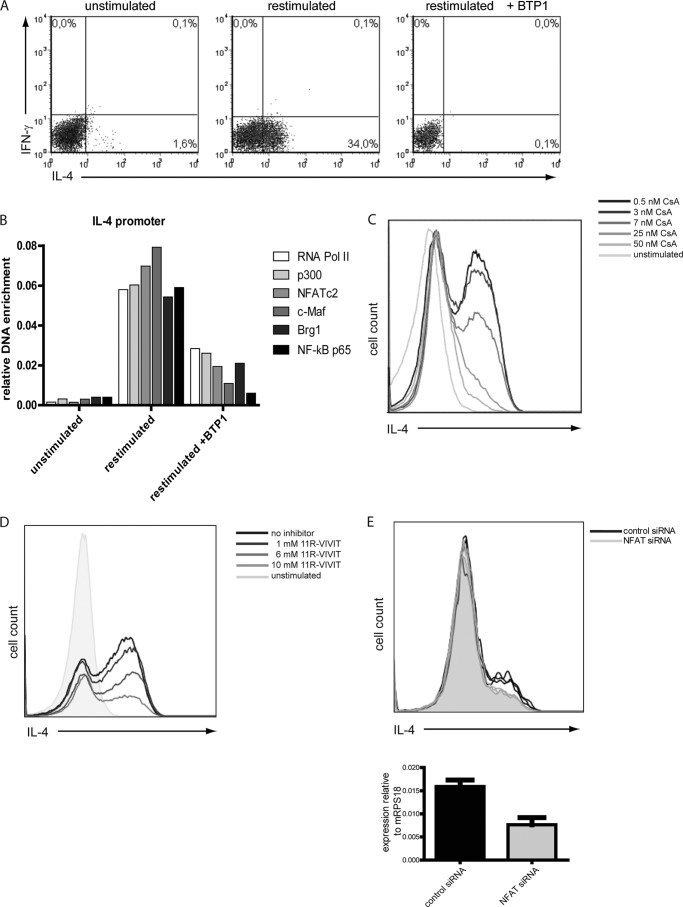
**Calcineurin activity digitalizes IL-4 expression in restimulated Th2 cells.** *A*, Th2 cells polarized for 12 days were restimulated for 3 h in the presence or absence of BTP1, selectively inhibiting the dephosphorylation of NFAT. The expression of IL-4 and IFNγ was determined by intracellular cytokine staining. *B*, Th2 cells restimulated for 3 h in the presence or absence of BTP1 were fixed. ChIP was performed against the indicated transcription factors and probed for binding to the *Il4* promoter. Values are representative of two independent experiments. *C* and *D*, Th2 cells were restimulated for 3 h in the presence of different concentrations of the calcineurin inhibitor CsA or the peptide inhibitor 11R-VIVIT and then stained intracellularly for IL-4 expression. Shown is an overlay of the histogram representation of the IL-4 staining. Shown are the representative results of six and three independent experiments, respectively. *E*, Th2 cells polarized for 6 days and restimulated with anti-CD3 and anti-CD28 antibodies. After 48 h, the cells were treated with NFATc2-specific and control siRNA. Four days later, the cells were restimulated with PMA/ionomycin for 3 h. mRNA was isolated to quantify NFATc2 knockdown efficiency, and expression of IL-4 was analyzed by intracellular staining. Results are representative of three independent experiments. *RNA Pol II*, RNA polymerase II.

Naive DO11.10 TCR transgenic CD4^+^ Th cells were stimulated under Th2-polarizing conditions for 12 days and then restimulated with PMA/ionomycin. IL-4 expression was assessed by intracellular cytokine staining showing that 34% of the Th2 cells reexpressed IL-4. When the NFATc2 dephosphorylation by calcineurin was selectively blocked by 25 nm of the specific inhibitor BTP1, a 3,5-bistrifluoromethyl pyrazole derivative (21), IL-4 reexpression was completely blocked (Fig. 4*A*). In those cells, binding of RNA polymerase II, p300, NFATc2, c-Maf, Brg1, and NF-κB p65 to the *Il4* promoter, 3 h after restimulation (Fig. 4*B*), was decreased to levels observed for IL-4-non-expressing Th2 cells (Fig. 3*D*). This shows that the dephosphorylation of NFATc2 by calcineurin is critical for the assembly of a transcriptional activator complex at the *Il4* gene.

Calcineurin, thus, translates graded differences in TCR signaling into an all-or-none expression of *Il4* of restimulated Th2 cells. This became evident when calcineurin was inhibited by CsA in different concentrations. Increasing CsA concentrations resulted in dose-dependent, decreased frequencies of IL-4 expressing Th2 cells following restimulation with PMA/ionomycin (Fig. 4*C*) or anti-CD3/CD28 antibodies (data not shown). However, the amount of IL-4 expressed by individual IL-4-expressing cells remained the same. As CsA has been described to also affect NF-κB activation (22), NFAT dephosphorylation was also blocked by the specific peptide inhibitor 11R-VIVIT (Fig. 4*D*) (23) and by specific siRNA targeting NFATc2 (Fig. 4*E*). Specific inhibition of either NFAT desphosphorylation by 11R-VIVIT or knockdown of NFATc2 itself by siRNA resulted in the reduction of the frequency of IL-4-expressing Th2 cells but not the amount of IL-4 expressed per cell.

##### IL-4 Expression Correlates with NFATc2 Nuclear Translocation

**FIGURE 5.**
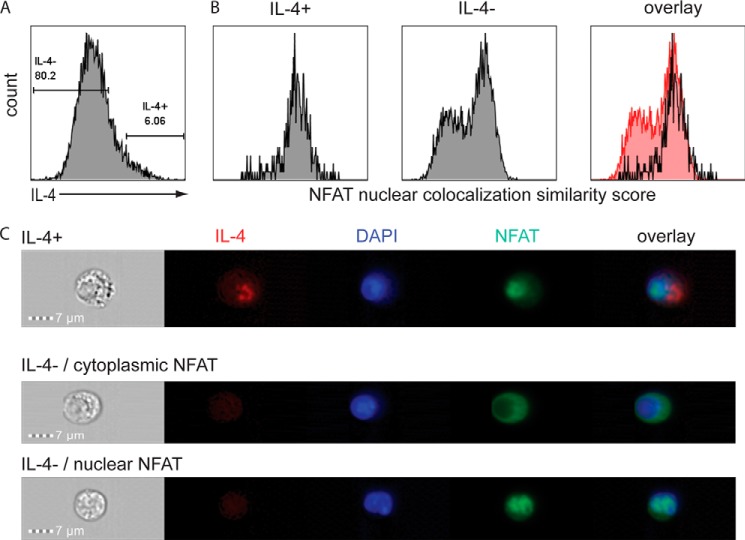
**NFATc2 translocates to the nucleus in IL-4 expressing Th2 cells.** *A*, Th2 cells polarized for 12 days were restimulated for 3 h in the presence of 7 nm CsA and stained intracellularly for IL-4 and NFATc2. The cells were analyzed with the image cytometer. Regions indicate IL-4-expressing and non-expressing Th2 cells. *B*, NFATc2 staining was correlated with nuclear DAPI staining in IL-4-expressing and non-expressing Th2 cells. A high similarity score indicates colocalization of NFATc2 with DAPI. *C*, exemplary images of Th2 cells stained for IL-4, NFATc2, and DAPI. Brightfield image, single stain images, and overlay of all stains for all combinations observed are shown.

To visualize the nuclear translocation of NFATc2 in Th2 cells on the single cell level, *in vitro*-generated Th2 cells were restimulated with PMA/ionomycin in the presence of 7 nm CsA and stained for NFATc2 and IL-4. The cells were analyzed by image cytometry. Among all IL-4-expressing Th2 cells (Fig. 5*A*), NFATc2 showed nuclear localization which was defined by a high similarity score representing the correlation coefficient between the NFATc2 fluorescent signal and the nuclear DAPI fluorescent signal (Fig. 5, *B* and *C*). Th2 cells that did not reexpress IL-4 showed a bimodal distribution having either only cytoplasmic or only nuclear NFATc2 (Fig. 4, *B* and *C*). Taken together, our data indicate that in individual Th2 cells, calcineurin, by cooperative activating dephosphorylation of NFATc2, digitalizes graded differences in TCR signaling into all-or-none decisions to express IL-4.

## DISCUSSION

Here, we show that in the TCR signaling cascade, calcineurin, by cooperative dephosphorylation of NFATc2, translates differences of signaling strength in individual, restimulated Th2 lymphocytes into an all-or-none decision to express or not the signature cytokine IL-4. NFAT is required to assemble at the *Il4* gene a transcription factor complex containing GATA-3, RNA polymerase II, NFATc2, NFATc1, NF-κB p65, c-Maf, CBP/p300, Brg1, and STAT6. Of these, only STAT6 and GATA-3 can bind in the absence of NFAT in Th2 cells not expressing IL-4.

Th2 lymphocytes are imprinted epigenetically by DNA demethylation and histone modification of the *Il4* gene (7, 24) and transcriptionally by expression of the lineage-determining transcription factor GATA-3 (5, 25), to express the signature cytokine IL-4 when restimulated by antigen. Surprisingly, and as noted early on, not all Th2 cells express IL-4 in a given restimulation (15). This is not a matter of lack of competence, as Th2 cells not expressing IL-4 in a given restimulation can express IL-4 in a subsequent restimulation at frequencies equal to cells that had expressed IL-4 (15). It has been speculated that the infidelity of IL-4 reexpression by individual Th2 cells might be due to stochastic, monoallelic expression of the *Il4* gene, with some cells not expressing it at all. It remained unclear, whether accessibility of the *Il4* gene (26–28) or availability of the transcription factors necessary for expression were the rate-limiting determinants. Moreover, in these studies, monoallelic expression of *Il4* was analyzed for genetically modified T lymphocytes that had one *Il4* allele marked by knock-in of a reporter gene, either green fluorescent protein (*Gfp*) (15) or *CD2* (29). For the *Gfp* knock-in Th lymphocytes, we have shown previously that the genetic insertion had replaced the GATA-3 binding site CIRE, disabling the epigenetic imprinting of the modified *Il4* allele (7). For wild-type Th2 cells, stochastic monoallelic expression of *Il4* would predict a subpopulation of cells expressing both alleles and consequently twice as much as the cells expressing only one allele. This was not observed.

Here, we show that reexpression of *Il4* by Th2 lymphocytes is not only due to stochastic variations but is determined by activation of NFATc2 by calcineurin. NFAT has 23 phosphorylation sites, which are dephosphorylated by calcineurin in a cooperative fashion, *i.e.* with strictly sigmoid kinetics, resulting in a “molecular switch” (30). NFAT has to be dephosphorylated at 13 of these sites to expose its nuclear translocation sequence. Dephosphorylation at serine residues at the N-terminal transactivation domain is required for NFAT to bind to its target DNA sequence (18). Translocation of NFATc2 into the nucleus of activated T lymphocytes, thus, is an all-or-none event (Fig. 5*B*) (17). For human Th lymphocytes, this results in all-or-none reexpression of IL-2, which is dependent on TCR signaling strength and mediated by calcineurin (17). Calcineurin and NFATc2, thus, qualify as molecular analog-to-digital converters, translating TCR signaling strength into different frequencies of cells expressing NFAT-dependent genes. In established Th effector/memory cells, it is the epigenetic imprint of a cell determining which genes are accessible, as we show here for murine Th2 lymphocytes.

Interestingly, independent of the frequency of IL-4-expressing cells in a given restimulation, the average amount of IL-4 expressed by the individual Th2 cell is the same, with stochastic cell-to-cell variability (31). This shows that under the conditions analyzed, none of the transcription factors required for *Il4* expression is rate-limiting, except NFAT, as is evident from selective inhibition by BTP1 (32), 11R-VIVIT (22), or siRNA. NFAT is required to assemble GATA-3, RNA polymerase II, NFATc2, NFATc1, NF-κB p65, c-Maf, CBP/p300, Brg1, and STAT6 at the regulatory regions of the *Il4* gene. The transcriptional activator complex may contain more proteins, which have not been analyzed here. STAT6 and GATA-3 did bind to the *Il4* gene also in the absence of NFAT in restimulated Th2 cells not expressing IL-4, and GATA-3 remained bound to the *Il4* gene in Th2 cells expressing IL-4 at late time points of restimulation. Apparently, on their own, they are not competent to assemble any of the other transcription factors analyzed to the *Il4* gene, in particular not CBP/p300 and Brg1, which have been connected to epigenetic imprinting (12, 33). Although GATA-3 itself has been shown to be critical for epigenetic imprinting of the *Il4* gene (26) and is the lineage-determining transcription factor of Th2 cells (5, 25), it is not required for the maintenance of the Th2 phenotype, with respect to IL-4 expression. Unlike inhibition of NFATc2 activity as shown here, conditional deletion of GATA-3 in already established Th2 cells did not change the frequency of Th2 cells reexpressing IL-4 but instead reduced the amount of IL-4 expressed per cell (6). In the Th2 cells analyzed here, GATA-3 expression obviously was not rate-limiting, as both Th2 cells, expressing IL-4 or not, expressed similar amounts of GATA-3.

The conversion of graded, analog differences in antigen receptor signal strength into expression of defined packages of cytokines in activated T lymphocytes by the calcineurin/NFAT switch, teaches us that in immune reactions, communication between individual cells via NFAT-dependent cytokines occurs in an all-or-none fashion, probably by direct contact and contact-directed secretion (34). This phenomenon is analogous to the signal transduction in neurons, where a stimulus leads to the opening of ion channels and the firing of an action potential. Increasing the strength of the stimulus does not increase the size of the action potential but rather increases the frequency of action potentials (35). This all-or-none principle ensures that neural signals are passed on in full strength once a certain threshold is passed.

Our data indicate that in adapting the magnitude of the immune response to different concentrations of antigens, it is the frequencies of responding cells among those able to respond, which is regulated by the calcineurin/NFAT switch. The advantage of this analog-to-digital conversion would be that the immune system is able to mount immune responses, even if by only a few cells, to antigens of low abundance, and it defines a threshold of reaction for the individual cell, minimizing background expression of potentially harmful genes, *i.e.* immunopathology.

## Footnotes

2 The abbreviations used are:

Th: T helper
CsA: cyclosporin A
BTP1: 3,5-bistrifluoromethyl pyrazole
CIRE: conserved intronic regulatory element
HSS: hypersensitivity site
NFAT: nuclear factor of activated T cells
STAT: signal transducer and activator of transcription
LCR: locus control region
PMA: phorbol 12-myristate 13-acetate
TCR: T cell receptor.
